# Training principles to advance expertise

**DOI:** 10.3389/fpsyg.2014.00131

**Published:** 2014-02-19

**Authors:** Alice F. Healy, James A. Kole, Lyle E. Bourne

**Affiliations:** ^1^Department of Psychology and Neuroscience, University of ColoradoBoulder, CO, USA; ^2^School of Psychological Sciences, University of Northern ColoradoGreeley, CO, USA

**Keywords:** training, acquisition, retention, transfer, efficiency, durability, generalizability, expertise

## Introduction

There are three forms of task engagement that are the basis of successful training for expertise—acquisition, retention, and transfer—and three corresponding goals of training—efficiency, durability, and generalizability. This paper reviews training conditions that facilitate acquisition, enhance retention, and promote transfer to contexts not encountered during training. Diligent practice under these training conditions can lead eventually to an elite level of performance or to expertise.

In this review of training principles, we emphasize those that are derived from work in our laboratory. We have found that developing training that optimizes efficiency, durability, and generalizability, however, is something of a balancing act because what promotes efficient training often comes at a price in durability, and durable training is not always generalizable (see Healy et al., 2012; Bourne and Healy, 2014). These tradeoffs are due in part to the fact that training might involve two different types of knowledge—declarative and procedural. Declarative knowledge consists of facts, whereas procedural knowledge consists of skills, or ways to use the facts, and these two types of knowledge differ in terms of their durability and generalizability.

## Acquisition

There are training principles that can be used to improve the efficiency of acquiring knowledge and skills. One way to increase training speed involves the *scheduling of feedback* given to the trainee. Trial-by-trial feedback can facilitate learning in many situations, especially by improving motivation and by identifying and correcting errors. Frequent feedback can be distracting, however, when the trainees already have a good sense of how well they are responding. In those cases periodic summary feedback, which is provided on only some percentage of training trials, can be a more effective procedure for promoting skill acquisition (Schmidt et al., 1989, demonstrated this principle with a ballistic timing task).

In terms of the difficulty of new material that is presented during training, there is an optimal *zone of learnability*, implying that training changes should be neither too difficult nor too easy. Specifically, training trials should contain information that is a little beyond what the trainee already knows or should require a bit faster or more accurate performance (Wolfe et al., 1998).

Training can be based on mental, as opposed to physical, practice. Although physical practice is better than mental practice in many circumstances, *mental practice* can be superior to physical practice when the practice involves different effectors from those used at testing (Wohldmann et al., 2008a). For example, training with the right hand and testing with the left hand can lead to interference during physical practice, but not during mental practice, which involves a more abstract representation of the skill that appears to be effector independent. Mental practice is especially useful, of course, when circumstances do not allow physical practice.

The *focus of attention* can be varied in training, and training usually benefits from an external focus on the results of body movements as opposed to an internal focus on the body movements themselves (Wulf, 2007). For example, to perfect the skill of dart throwing, attention should be focused on the trajectory of the dart rather than on the motion of the arm (Lohse et al., 2010).

Research has concluded that for learning new skills (but not necessarily for learning new facts) rest intervals should be interpolated between practice trials (distributed practice) rather then practicing without rest intervals (massed practice), especially if testing occurs after a delay following practice (see Cepeda et al., 2006, for a review of effects involving *spacing of practice*).

When engaged in prolonged work on a routine task, it is often advisable to introduce into the training regimen a *cognitive antidote*, which is a simple cognitive requirement that can be added to minimize task disengagement and boredom and to mitigate a decline in accuracy across trials (Kole et al., 2008). For example, when entering into a computer a long sequence of numbers, alternating between using the + and − keys as the concluding keystroke, rather than using only a single key to conclude each number, increases the accuracy of digit entry and eliminates the usual increase in errors that occurs with increasing practice.

Task requirements can sometimes be broken up into parts, with practice at the beginning of training involving only one or more parts of a task rather than the whole task (Wickens et al., 2012). The recommendation to use *part-task training* applies to a segmented task, when the parts are performed sequentially (Wightman and Lintern, 1985), but not to a fractionated task, when the parts are performed simultaneously (Adams and Hufford, 1962).

## Retention

The durability of training can be enhanced by selected training principles. Some of these principles benefit training retention with a cost in training efficiency, but others benefit retention with little or no cost in training efficiency.

### Retention variables with a cost in efficiency

Complications can sometimes be added to training to promote retention (Schneider et al., 2002). However, not all complications that increase *task difficulty* are desirable (Bjork, 1994); difficulties at training facilitate retention only when they force learners to apply task-relevant cognitive processes (McDaniel and Butler, 2011). For example, in training RADAR detection, adding to training a secondary task that was irrelevant lowered RADAR detection performance at test, but adding a relevant secondary task at training aided RADAR detection accuracy at test (Young et al., 2011).

As research on experts has shown (Ericsson et al., 1980), training should make *strategic use of knowledge* that trainees already have (Kole and Healy, 2007). For example, when learning facts about unknown people, associating each of those people with someone familiar (e.g., a friend or relative) requires additional learning but can considerably enhance acquisition and retention of those facts.

Also following the methods of experts, training can be improved by using *memory strategies*, such as the keyword method, by which two items can be associated in memory through a keyword serving as a mediating link (Kole and Healy, 2013). When learning the association between the French word *jambe* and the English word *leg*, for example, learners could use the keyword *jam* and form an interactive image of some sticky jam on someone's leg. This method will allow the learners to derive the translation of *jambe*, but it does require forming extra links from *jambe* to *jam* and from *jam* to *leg*, which slows initial acquisition.

### Retention variables with no cost in efficiency

Items can be grouped together in chunks during training to promote retention. *Item chunking* yields no loss, and probably a gain, in training efficiency (Miller, 1956). For example, when required to type a four-digit number (e.g., 9632), individuals often find it helpful to represent the number as two two-digit chunks (96 and 32) (Fendrich et al., 1991). Also, memory experts can learn a long list of digits by dividing them into chunks representing familiar sequences like running times or dates (Ericsson et al., 1980).

Similar to chunking, two separate tasks can often be combined into a single *functional task* to promote durability with no cost to efficiency. If a secondary task is combined with a primary task at test, the two tasks should be combined during training as well for best test performance (Wohldmann et al., 2012). In fact, in some cases removing at test a difficult secondary task that was available during training can retard test performance; for example, removing an alphabet-counting task used during the training of time estimation can increase errors in time estimation during test (Healy et al., 2005).

For optimal retention, the procedures used in training should be duplicated at test. *Procedural reinstatement* is effective because declarative information (facts) shows strong generalizability but weak durability, whereas procedural information (skills) shows strong durability but weak generalizability (Lohse and Healy, 2012). Despite the high degree of transfer for declarative information, when learning facts it is best to make sure that there is an overlap in the processing required at training and test (i.e., ensure that there is transfer appropriate processing; Morris et al., 1977; Roediger et al., 1989). Another way to improve the retention of factual material is by studying it at its most meaningful level, as opposed to studying it at a superficial level (e.g., paying attention to the sound or spelling of the material), an effect called *depth of processing* (Craik and Lockhart, 1972).

Practice retrieving factual information, instead of simply restudying the material, can improve retention, demonstrating a *testing effect* (Roediger and Karpicke, 2006). A related method to enhance fact retention is to generate the information rather than just to read it, demonstrating a *generation effect* (Slamecka and Graf, 1978). For example, if the task is to remember the answers to a set of multiplication problems, it is better to generate or verify the answers to the problems than to simply read them or perform the multiplication using a calculator (Crutcher and Healy, 1989). See Karpicke and Zaromb (2010) for a comparison of the similar but not identical benefits of testing and generation.

For skills, periodic restudy of material during periods of disuse, called *refresher training*, enhances skill retention (McDaniel, 2012). On the other hand, training should not be done more than necessary, resulting in *overlearning or overtraining*, because extra practice produces diminishing performance enhancement returns (Driskell et al., 1992).

## Transfer

Improving the generalizabilty of knowledge and skills is considerably more difficult than improving their efficiency and durability. There are only a few training principles shown to be effective for enhancing task transfer.

One way to enhance transfer is to change the conditions of practice periodically, thereby increasing the *variability of practice* (Schmidt and Bjork, 1992). Not all forms of variable practice are effective, however. Varying parameters within a single generalized motor program can enhance transfer, but varying the generalized motor programs themselves might not benefit transfer. For example, learning how to cope with a variety of defective computer mice that vary in their properties (e.g., a mouse that reverses only vertical movements and another mouse that reverses both horizontal and vertical movements) does not enhance transfer to a new type of defective mouse (e.g., a mouse that reverses only horizontal movements) (Healy et al., 2006). Practicing to move a single defective mouse to a variety of targets, however, does enhance transfer to moving that same mouse to new targets (Wohldmann et al., 2008b).

For tasks involving quantitative estimation (e.g., of country populations or intercity distances), a technique of *seeding the knowledge base* can be used, in which a few selected examples are given that define a domain and thereby enhance transfer to unpracticed examples (Brown and Siegler, 1996).

When possible, the discovery of rules can lead to generalizable knowledge, so that searching for systematic relationships among examples can bolster transfer of training, showing the advantage for *rules* vs*. instance memory* (Bourne et al., 2010; McDaniel et al., in press). For example, in learning how to choose between the alternate pronunciations for the word *the* (as thuh or thee) individuals can either learn the pronunciation preceding every single word or can learn the rule that thuh is used preceding words starting with a consonant sound and thee is used preceding words starting with a vowel sound.

## Concluding remarks

This summary, which is outlined in Table 1, does not include all training principles that have been shown to be effective in promoting training efficiency, durability, and generalizability. For additional principles, readers are referred to Bourne and Healy (2014) and Healy et al. (2012), and for an initial attempt to account for the principles in a single theoretical framework, readers are referred to Jones et al. (2012), which is available by request from the corresponding author.

**Table 1:** **Training principles as a function of form of task engagement and training goal**.

**ACQUISITION, EFFICIENCY**
Scheduling of feedback
Zone of learnability
Mental practice
Focus of attention
Spacing of practice
Cognitive antidote
Part-task training
**RETENTION, DURABILITY**
*Retention variables with a cost in efficiency*
Task difficulty
Strategic use of knowledge
Memory strategies
*Retention variables with no cost in efficiency*
Item chunking
Functional task
Procedural reinstatement
Depth of processing
Testing effect
Generation effect
Refresher training
Overlearning or overtraining
**TRANSFER, GENERALIZABILITY**
Variability of practice
Seeding the knowledge base
Rules vs. instance memory

Almost all of the studies reviewed here have involved novice learners. Nevertheless, these same principles should apply for training at any level, so they should be informative with respect to the issue of training to an elite level of performance. In fact, some of these principles (e.g., focus of attention) seem to apply more strongly for experts than for novices (e.g., Perkins-Ceccato et al., 2003). To reach elite levels of performance, however, the learners need to couple these training principles with the use of deliberate, highly focused, and effortful practice (Ericsson et al., 1993).

## Author contributions

Alice F. Healy wrote the initial and final drafts of the manuscript. James A. Kole and Lyle E. Bourne Jr. edited the initial draft of the manuscript and suggested revisions of it.

## References

[B1] Adams J. A. Hufford L. E. (1962). Contributions of a part-task trainer to the learning and relearning of a time-shared flight maneuver. Hum. Factors 4, 159–170

[B2] Bjork R. A. (1994). Memory and metamemory considerations in the training of human beings, in Metacognition: Knowing About Knowing, eds Metcalfe J. Shimamura A. (Cambridge, MA: MIT Press), 185–205

[B3] Bourne L. E. Jr. Healy A. F. (2014). Train Your Mind for Peak Performance: A Science-Based Approach for Achieving Your Goals. Washington, DC: American Psychological Association

[B4] Bourne L. E. Jr. Raymond W. D. Healy A. F. (2010). Strategy selection and use during classification skill acquisition. J. Exp. Psychol. Learn. Mem. Cogn. 36, 500–514 10.1037/a0018599 20192545

[B5] Brown N. R. Siegler R. S. (1996). Long-term benefits of seeding the knowledge base. Psychol. Bull. Rev. 3, 385–388 10.3758/BF03210766 24213943

[B6] Cepeda N. J. Pashler H. Vul E. Wixted J. T. Rohrer D. (2006). Distributed practice in verbal recall tasks: a review and quantitative synthesis. Psychol. Bull. 132, 354–380 10.1037/0033-2909.132.3.354 16719566

[B7] Craik F. I. M. Lockhart R. S. (1972). Levels of processing: a framework for memory research. J. Verbal Learn. Verbal Behav. 11, 671–684 10.1016/S0022-5371(72)80001-X

[B8] Crutcher R. J. Healy A. F. (1989). Cognitive operations and the generation effect. J. Exp. Psychol. Learn. Mem. Cogn. 15, 669–675 10.1037/0278-7393.15.4.669

[B9] Driskell J. E. Willis R. P. Copper C. (1992). Effect of overlearning on retention. J. Appl. Psychol. 77, 615–622 10.1037/0021-9010.77.5.615

[B10] Ericsson K. A. Chase W. G. Faloon S. (1980). Acquisition of a memory skill. Science 208, 1181–1182 10.1126/science.7375930 7375930

[B11] Ericsson K. A. Krampe R. T. Tesch-Römer C. (1993). The role of deliberate practice in the acquisition of expert performance. Psychol. Rev. 100, 363–406 10.1037/0033-295X.100.3.363

[B12] Fendrich D. W. Healy A. F. Bourne L. E. Jr. (1991). Long-term repetition effects for motoric and perceptual procedures. J. Exp. Psychol. Learn. Mem. Cogn. 17, 137–151 10.1037/0278-7393.17.1.137 1826728

[B13] Healy A. F. Schneider V. I. Bourne L. E. Jr. (2012). Empirically valid principles of training, in Training Cognition: Optimizing Efficiency, Durability, and Generalizability, eds Healy A. F. Bourne L. E. Jr. (New York, NY: Psychology Press), 13–39

[B14] Healy A. F. Wohldmann E. L. Parker J. T. Bourne L. E. Jr. (2005). Skill training, retention, and transfer: the effects of a concurrent secondary task. Mem. Cogn. 33, 1457–1471 10.3758/BF03193378 16615393

[B15] Healy A. F. Wohldmann E. L. Sutton E. M. Bourne L. E. Jr. (2006). Specificity effects in training and transfer of speeded responses. J. Exp. Psychol. Learn. Mem. Cogn. 32, 534–546 10.1037/0278-7393.32.3.534 16719664

[B16] Jones M. Bourne L. E. Jr. Healy A. F. (2012). A compact mathematical model for predicting the effectiveness of training, in Training Cognition: Optimizing Efficiency, Durability, and Generalizability, eds Healy A. F. Bourne L. E. Jr. (New York, NY: Psychology Press), 247–266

[B17] Karpicke J. D. Zaromb F. M. (2010). Retrieval mode distinguishes the testing effect from the generation effect. J. Mem. Lang. 62, 227–239 10.1016/j.jml.2009.11.010

[B18] Kole J. A. Healy A. F. (2007). Using prior knowledge to minimize interference when learning large amounts of information. Mem. Cognit. 35, 124–137 10.3758/BF03195949 17533887

[B19] Kole J. A. Healy A. F. (2013). Is retrieval mediated after repeated testing? J. Exp. Psychol. Learn. Mem. Cogn. 39, 462–472 10.1037/a0028880 22686841

[B20] Kole J. A. Healy A. F. Bourne L. E. Jr. (2008). Cognitive complications moderate the speed-accuracy tradeoff in data entry: a cognitive antidote to inhibition. Appl. Cogn. Psychol. 22, 917–937 10.1002/acp.1401

[B21] Lohse K. R. Healy A. F. (2012). Exploring the contributions of declarative and procedural information to training: a test of the procedural reinstatement principle. J. Appl. Res. Mem. Cogn. 1, 65–72 10.1016/j.jarmac.2012.02.002

[B22] Lohse K. R. Sherwood D. E. Healy A. F. (2010). How changing the focus of attention affects performance, kinematics, and electromyography in dart throwing. Hum. Mov. Sci. 29, 542–555 10.1016/j.humov.2010.05.001 20541275

[B23] McDaniel M. A. (2012). Put the SPRINT in knowledge training: training with SPacing, Retrieval, and INTerleaving, in Training Cognition: Optimizing Efficiency, Durability, and Generalizability, eds Healy A. F. Bourne L. E. Jr. (New York, NY: Psychology Press), 267–286

[B24] McDaniel M. A. Butler A. C. (2011). A contextual framework for understanding when difficulties are desirable, in Successful Remembering and Successful Forgetting: A Festschrift in Honor of Robert A. Bjork, ed Benjamin A. S. (New York, NY: Psychology Press), 175–198

[B25] McDaniel M. A. Cahill M. J. Robbins M. Wiener C. (in press). Individual differences in learning and transfer: stable tendencies for learning exemplars versus abstracting rules. J. Exp. Psychol. Gen. 10.1037/a0032963 23750912 PMC3890377

[B26] Miller G. A. (1956). The magical number seven, plus or minus two: some limits on our capacity for processing information. Psychol. Rev. 63, 81–97 10.1037/h0043158 13310704

[B27] Morris C. D. Bransford J. D. Franks J. J. (1977). Levels of processing versus transfer appropriate processing. J. Verbal Learn. Verbal Behav. 16, 519–533 10.1016/S0022-5371(77)80016-919882435

[B28] Perkins-Ceccato N. Passmore S. R. Lee T. D. (2003). Effects of focus of attention depend on golfers' skill. J. Sport Sci. 21, 593–600 10.1080/0264041031000101980 12875310

[B29] Roediger H. L. III. Karpicke J. D. (2006). The power of testing memory: basic research and implications for educational practice. Perspect. Psychol. Sci. 1, 181–210 10.1111/j.1745-6916.2006.00012.x 26151629

[B30] Roediger H. L. III. Weldon M. S. Challis B. H. (1989). Explaining dissociations between implicit and explicit measures of retention: a processing account, in Varieties of Memory and Consciousness: Essays in Honour of Endel Tulving, eds Roediger H. L. III. Craik F. I. M. (Hillsdale, NJ: Erlbaum), 3–41

[B31] Schmidt R. A. Bjork R. A. (1992). New conceptualizations of practice: common principles in three paradigms suggest new concepts for training. Psychol. Sci. 3, 207–217 10.1111/j.1467-9280.1992.tb00029.x

[B32] Schmidt R. A. Young D. E. Swinnen S. Shapiro D. C. (1989). Summary knowledge of results for skill acquisition: support for the guidance hypothesis. J. Exp. Psychol. Learn. Mem. Cogn. 15, 352–359 10.1037/0278-7393.15.2.352 2522520

[B33] Schneider V. I. Healy A. F. Bourne L. E. Jr. (2002). What is learned under difficult conditions is hard to forget: contextual interference effects in foreign vocabulary acquisition, retention, and transfer. J. Mem. Lang. 46, 419–440 10.1006/jmla.2001.2813

[B34] Slamecka N. J. Graf P. (1978). The generation effect: delineation of a phenomenon. J. Exp. Psychol. Hum. Learn. Mem. 4, 592–604 10.1037/0278-7393.4.6.592

[B35] Wickens C. Hutchins S. Carolan T. Cumming J. (2012). Attention and cognitive resource load in training strategies, in Training Cognition: Optimizing Efficiency, Durability, and Generalizability, eds Healy A. F. Bourne L. E. Jr. (New York, NY: Psychology Press), 67–88

[B36] Wightman D. Lintern G. (1985). Part-task training for tracking and manual control. Hum. Factors 27, 267–283 11540255

[B37] Wohldmann E. L. Healy A. F. Bourne L. E. Jr. (2008a). A mental practice superiority effect: less retroactive interference and more transfer than physical practice. J. Exp. Psychol. Learn. Mem. Cogn. 34, 823–833 10.1037/0278-7393.34.4.823 18605871

[B38] Wohldmann E. L. Healy A. F. Bourne L. E. Jr. (2008b). Global inhibition and midcourse corrections in speeded aiming. Mem. Cognit. 36, 1228–1235 10.3758/MC.36.7.1228 18927039

[B39] Wohldmann E. L. Healy A. F. Bourne L. E. Jr. (2012). Specificity and transfer effects in time production skill: examining the role of attention. Atten. Percept. Psychophys. 74, 766–778 10.3758/s13414-012-0272-5 22302632

[B40] Wolfe M. B. W. Schreiner M. E. Rehder B. Laham D. Foltz P. W. Kintsch W. (1998). Learning from text: matching readers and text by latent semantic analysis. Discourse Process. 25, 309–336 10.1080/01638539809545030

[B41] Wulf G. (2007). Attention and Motor Skill Learning. Champaign, IL: Human Kinetics

[B42] Young M. D. Healy A. F. Gonzalez C. Dutt V. Bourne L. E. Jr. (2011). Effects of training with added difficulties on RADAR detection. Appl. Cogn. Psychol. 25, 395–407 10.1002/acp.1706

